# Periodontal Disease and Risk of Preeclampsia: A Meta-Analysis of Observational Studies

**DOI:** 10.1371/journal.pone.0070901

**Published:** 2013-08-12

**Authors:** Ben-Juan Wei, Yi-Jun Chen, Li Yu, Bin Wu

**Affiliations:** 1 Department of Stomatology, Ren Ji Hospital, School of Medicine, Shanghai Jiao Tong University, Shanghai, China; 2 Department of Pharmacy, Ren Ji Hospital, School of Medicine, Shanghai Jiao Tong University, Shanghai, China; University of Toronto, Canada

## Abstract

**Background:**

Many epidemiological studies have found a positive association between periodontal disease (PD) and the risk of preeclampsia, but the magnitude of this association varies and independent studies have reported conflicting findings. We performed a meta-analysis to ascertain the relationship between PD and preeclampsia.

**Methods:**

The PubMed database was searched up to January 12, 2013, for relevant observational studies on an association between PD and the risk of preeclampsia. Data were extracted and analyzed independently by two authors. The meta-analysis was performed using comprehensive meta-analysis software.

**Results:**

Thirteen observational case-control studies and two cohort studies, involving 1089 preeclampsia patients, were identified. Based on a random-effects meta-analysis, a significant association between PD and preeclampsia was identified (odds ratio = 2.79, 95% confidence interval CI, 2.01–3.01, P<0.0001).

**Conclusions:**

Although the causality remains unclear, the association between PD and preeclampsia may reflect the induction of PD by the preeclamptic state, or it may be part of an overall exaggerated inflammatory response to pregnancy. Larger randomized controlled trials with preeclampsia as the primary outcome and pathophysiological studies are required to explore causality and to dissect the biological mechanisms involved.

## Introduction

Periodontal disease (PD) is a chronic destructive inflammatory disease affecting the tooth-supporting tissues, and it is one of the most prevalent chronic infections in humans. At least 35% of dentate adults aged between 30 and 90 years in the United States experience PD [1], and PD may affect up to 90% of the global population [2]. PD is initiated by oral microorganisms, but it is thought that severe periodontal breakdown is mediated by the inflammatory response of the host. Additionally, the inflammatory response may not be limited to the periodontal region. It is well documented that periodontal diseases can affect systemic illness, including atherosclerotic cardiovascular disease, diabetes, adverse pregnancy outcomes and chronic obstructive pulmonary disorder [3]–[9]. Preeclampsia is a maternal multi-organ disease that clinically manifests in the second half of pregnancy with the appearance of hypertension and proteinuria. It is a disorder unique to pregnancy, with a prevalence of approximately 2–3%, and it is one of the leading causes of maternal morbidity and mortality in the Western world [10]. Women with diseases associated with chronic low-grade inflammation, such as diabetes mellitus, hypertension, obesity and arterial diseases, have an increased risk of developing preeclampsia [11]. Because periodontal disease is also associated with low-grade inflammation, it can be hypothesized that patients with periodontal disease may have an increased risk of developing preeclampsia. Many epidemiological studies have found a positive association between PD and preeclampsia [12]–[16]. However, different studies have used different measurement methods and investigated different populations. Therefore, the magnitude of the association has varied, and different studies have also reported conflicting findings. Thus, the possible role of PD in the pathogenesis of preeclampsia remains an important but unresolved issue.

An improved understanding of this issue may have important public health and clinical implications given the possibility that preventing and treating PD may reduce the incidence of preeclampsia. The objectives of this study were (1) to evaluate the inconsistent results of published observational studies on the association between PD and the risk of preeclampsia by conducting a meta-analysis and (2) to gain a more robust understanding of the association between PD and the risk of preeclampsia.

## Methods

### Literature Search

We initially identified publications that investigated the association between PD and the risk of preeclampsia by searching the PubMed and Embase databases from their inceptions through February 10, 2013. The following search terms were used: (1) “periodontal disease” or “periodontitis” or “periodontal” or “gum disease” or “periodontium”, and (2) “pregnancy outcomes” or “pregnancy complications” or “preeclampsia” or “pregnancy hypertension”. The resulting papers were first screened by title and abstract. Full-text papers were obtained when the studies fulfilled the selection criteria, as described below. Full-text analyses were independently performed by two reviewers. Case reports, letters, reviews, abstract-only studies and commentaries were excluded from the search.

### Study Selection

We included any study that met all of the following criteria: (1) the study had a cross-sectional, case-control or cohort design; (2) clear diagnostic criteria for PD and preeclampsia were established; (3) the association between PD and the risk of preeclampsia was investigated; and (4) the odds ratios (ORs)/risk ratio (RR, for cohort studies) and the corresponding 95% confidence intervals (CIs), or the number of events required to calculate them, were reported. Two authors independently evaluated the eligibility of all studies retrieved from the databases. Disagreements were resolved by discussion or in consultation with a third author.

### Data Extraction and Quality Assessment for Included Studies

Two reviewers independently extracted data regarding the characteristics of each study using a standardized data collection form. Data were recorded as follows: first author’s last name, year of publication and country of origin; number of participants with PD and total number of participants; ascertainment of PD; assessment of preeclampsia; and statistical adjustments for confounding factors. Any disagreements were resolved by consensus.

The quality of each included study was independently evaluated by the three authors using the Newcastle-Ottawa Scale (NOS) [17]. In our analysis, studies of low, intermediate and high quality were defined with NOS scores of 1–3, 4–6, and 7–9, respectively.

### Statistical Analysis

The pooled OR with 95% CI between PD and preeclampsia was used to estimate the effect sizes. We conducted a stratified analysis for the study design. Statistical heterogeneity among the studies was assessed using Cochran’s Q test and the I^2^ statistic [18]. P<0.10 for the Q test or P>50% for I^2^ was considered statistically significant for heterogeneity. The fixed- and random-effect models were adapted to calculate the pooled estimate where appropriate. To assess whether publication bias may have impacted the statistical results, a funnel plot was created, and Egger’s test was performed [19], [20]. For Egger’s test, P<0.10 was considered to be statistically significant. All statistical tests were two-sided. All statistical analyses were conducted using R version 2.15.1 for Windows (The R Foundation for Statistical Computing, Vienna, Austria).The current analysis was performed according to the Meta-Analysis Of Observational Studies in Epidemiology (MOOSE) guidelines for meta-analysis of observational studies and the Preferred Reporting Items for Systematic Reviews and Meta-Analyses (PRISMA) guidelines [21], [22].

## Results

### Characteristics of the Subjects in the Selected Studies

**Figure 1:**
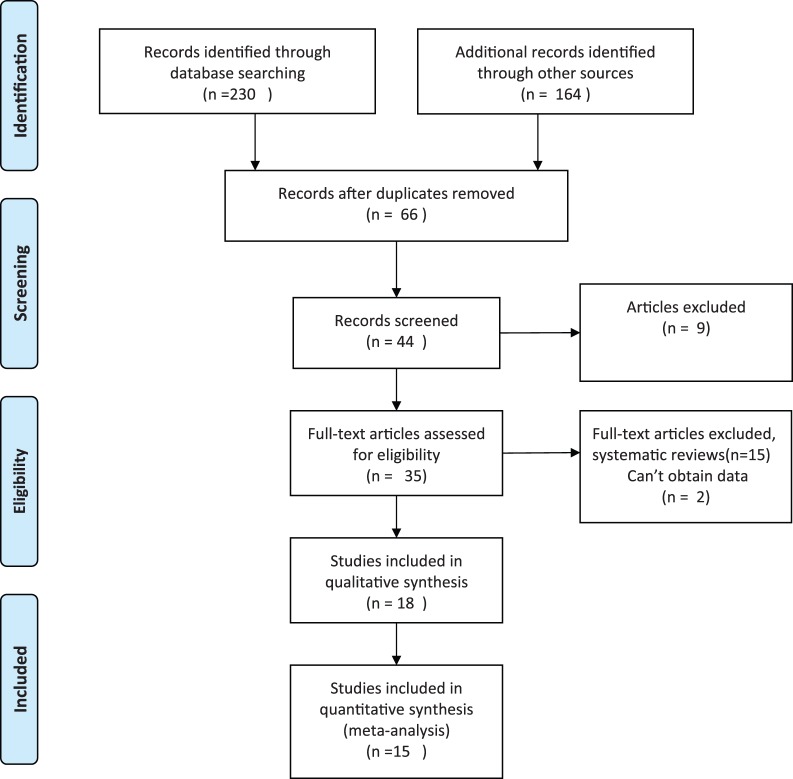
Flow chart of the literature search and article selection.

The detailed search procedures are summarized in Figure 1. The full text of the 18 identified articles was retrieved for detailed evaluation. Three of these articles were further excluded because they did not meet the inclusion criteria, including one study with univariate analyses only [23] and two studies that investigated hypertension only during pregnancy [24], [25]. Finally, the remaining 15 independent studies were used in the current analysis. Of these studies, 13 case control studies [12]–[16], [26]–[33] and two cohort studies measured the RRs for preeclampsia incidence [10], [34] (Table 1). Of these studies, three were conducted in North America, three in Europe, four in Asia, and five in South America. According to the NOS, 10 studies were of high quality and five were of intermediate quality. Except for four studies in which only demographics were adjusted for, the identified studies reported the effects after adjustment for other variables.

**Table 1:** Summary information for the included studies.

Reference	Design	Location	Group	Subjects	Assessmentof PD	Quality scaling (NOS)	Adjustment for covariates	OR (95% CI)
Boggess et al., 2003	cohort	USA	Preeclampsia	39	PI, CAL	9/9	1,3,5,6	2.1(1.0,4.4)
			Control	724				
Castaldi et al., 2006	cohort	Argentina	Preeclampsia	48	PI	6/9	1,3	0.99(0.70–1.40)
			Control	475				
Moura et al., 2012	case-control	Brazil	Preeclampsia	284	PI, CAL	8/9	1,2,3,4,5,7	2.03(1.43,2.90)
			Control	290				
Taghzouti et al., 2012	case-control	Canada	Preeclampsia	92	PI, CAL	6/9	1,2,4,5	1.13(0.59,2.17)
			Control	245				
Politano et al., 2011	case-control	Brazil	Preeclampsia	58	PI	7/9	1,2,5	3.73(1.32,10.58)
			Control	58				
Ha et al., 2011	case-control	Korea	Preeclampsia	16	CAL	7/9	7	6.60(1.25,41.61)
			Control	48				
Sayar et al., 2011	case-control	Iran	Preeclampsia	105	PI, CAL,	7/9	NA	4.1(1.5,11.5)
			Control	105				
Shetty et al., 2010	case-control	India	Preeclampsia	30	PI, CAL	8/9	NA	5.78(2.41,13.89
			Control	100				
Siqueira et., al 2008	case-control	Brazil	Preeclampsia	125	PI, CAL	5/9	1,5	1.52(1.01,2.29)
			Control	375				
Canakci et al., 2007	case-control	Turkey	Preeclampsia	38	PI, CAL	7/9	1,2,3,4,5	2.43(1.13,8.19)
			Control	21				
Kunnen et al., 2007	case-control	Netherlands	Preeclampsia	17	PI, CAL	7/9	1,4,5	7.9(1.9,32.8)
			Control	35				
Cota et al., 2006	case-control	Brazil	Preeclampsia	588	PI, CAL	6/9	1,4,5	1.88(1.1,3.0)
			Control	190				
Contreras et al., 2006	case-control	USA	Preeclampsia	130	CAL	6/9	NA	3.0(1.91,4.87)
			Control	243				
Canakci et al., 2004	case-control	Turkey	Preeclampsia	41	PI, CAL	9/9	NA	3.47(1.07,11.95)
			Control	41				
Lohsoonthorn et al., 2009	case-control	Thailand	Preeclampsia	150	PI, CAL	6/9	1,2,4,5,7	0.92(0.26–3.28)

NA, not available; ABL, alveolar bone loss; PI, periodontal index; CAL, clinical attachment loss; b: 1, Smoking; 2, body weight; 3, socioeconomic status; 4, education level; 5, age; 6, race; and 7, health behavior.

### Main Analysis

**Figure 2:**
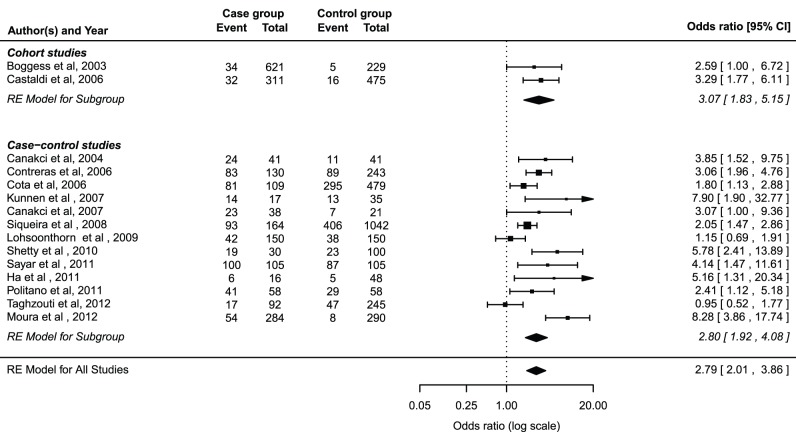
Forest plots for the meta-analysis of the association between PD and preeclampsia.

The pooled ORs from the 13 case control studies and two cohort studies are shown in Figure 2. The meta-analysis of the 15 studies suggested a positive association between PD and preeclampsia (summary OR = 2.79, 95% CI = 2.01 to 3.01) with significant heterogeneity among these studies (Q = 41.19, P = 0.0002, I^2^ = 69.75%).

A subgroup meta-analysis was performed by study design (Figure 2). A positive association was detected in the cohort studies (pooled OR = 3.07, 95% CI, 1.83 to 5.15), which was consistent with the estimated effect size in the case-control studies (pooled OR = 2.80, 95% CI, 1.92 to 4.08).

### Publication Bias

**Figure 3:**
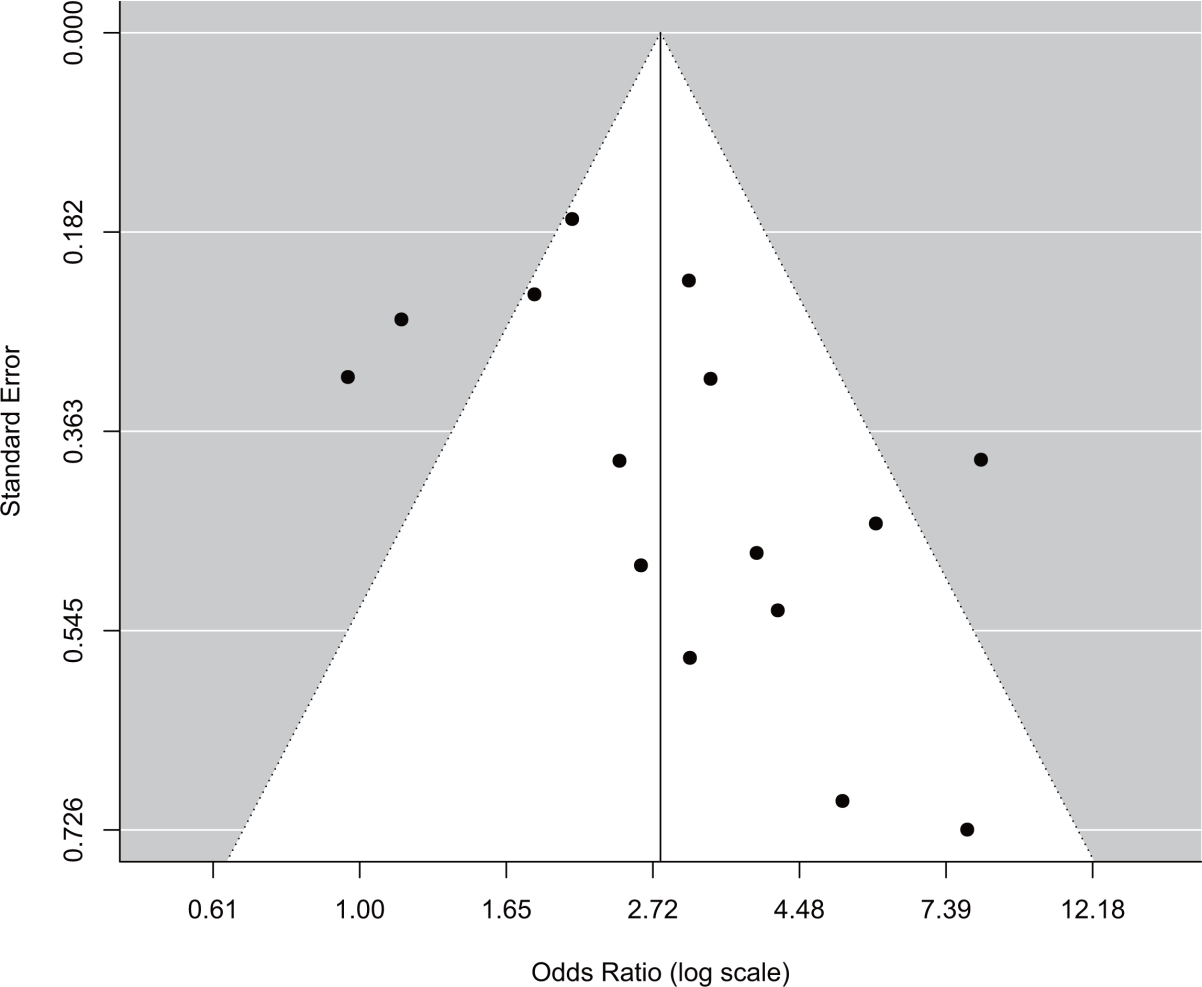
Funnel plot for all studies evaluating the association between PD and preeclampsia. Begg’s regression asymmetry test (P = 0.14).

Begg’s funnel plot for the association between PD and preeclampsia did not show the asymmetry that is typically associated with publication bias; the *P* value for Egger’s regression asymmetry test was 0.14, which indicates a low probability of publication bias (Figure 3).

## Discussion

Preeclampsia is a pregnancy-specific disorder that is characterized by an increase in systolic arterial pressure (≥140 mmHg) and/or diastolic pressure (≥90 mmHg) and proteinuria (≥300 mg/24 h), after 20 weeks of gestation. This condition is potentially dangerous for both the mother and the fetus. PD is considered a novel risk factor for preeclampsia, in addition to *Chlamydia pneumoniae* infection, HIV infection and malaria. Our meta-analysis of 13 case-control studies and two cohort studies provides evidence that PD is associated with a 2.79-fold increased risk of preeclampsia.

Periodontal disease is initiated by oral microorganisms, but it is believed that severe periodontal breakdown is mediated by the inflammatory response of the host. The inflammatory response may not be limited to the periodontal area. It has been proposed that daily episodes of bacteremia or dissemination of bacterial endotoxins from the periodontal focus may induce systemic activation of the inflammatory response. Bacteria or bacterial endotoxins in the systemic circulation may induce pro-inflammatory cytokine production. These cytokines then further activate the inflammatory response, which results in a chronic low-grade systemic up-regulation of the inflammatory molecules involving IL-6 and C-reactive protein (CRP) [35], [36]. The inflammatory response also activates inflammatory and endothelial cells and may result in endothelial dysfunction. In pregnancy, the immune response plays a pivotal role in maintaining a healthy equilibrium between the mother and fetal allograft. During a normal pregnancy, the specific immune response is shifted towards a Th2-type immune response, and the inflammatory response is also activated [37]. This activation of the inflammatory response during pregnancy is characterized by the increased expression of activation markers on monocytes and granulocytes, differences in monocyte cytokine production and increased circulating levels of pro-inflammatory cytokines and inflammatory markers, such as CRP.

Our meta-analysis has several strengths. First, many cases were included, which provides solid evidence for evaluating the epidemiologic association between PD and preeclampsia risk. Second, the included studies were conducted in different countries, which makes the results more generalizable. Third, based on the NOS, all studies included in this meta-analysis were of high or intermediate quality.

Our meta-analysis has several limitations. First, cohort studies and case-control studies may be susceptible to detection bias, and recall and selection biases are often present in case-control studies. Second, as the studies included in this meta-analysis were all observational studies, the observed positive association between PD and the risk of preeclampsia may have resulted from unmeasured factors. The only slight attenuation of effect size upon adjustment for age, body mass index (BMI)/obesity, smoking and alcohol consumption suggests that the association between PD and preeclampsia risk is robust and less likely to be greatly attenuated by unmeasured factors, although the residual confounding effects of the measured variables, particularly BMI/obesity, may not be completely removed. Fourth, none of the 15 studies selected in our meta-analysis provided the degree of PD and risk of preeclampsia; therefore, we were unable to conduct a dose-response analysis to assess the relationship between these variables more precisely. Fifth, the prevalences/incidences of preeclampsia in developing countries (where access to dental care is limited) are presumably much higher than those in developed countries (where access to dental care is better), but we could not obtain current relevant data to verify this assumption.

In summary, our meta-analysis suggests that PD may increase the risk of preeclampsia. Further investigations should assess the underlying biological links between PD and preeclampsia.
